# HIF1α and HIF2α Exert Distinct Nutrient Preferences in Renal Cells

**DOI:** 10.1371/journal.pone.0098705

**Published:** 2014-05-30

**Authors:** Alexandra Arreola, C. Lance Cowey, Jonathan L. Coloff, Jeffrey C. Rathmell, W. Kimryn Rathmell

**Affiliations:** 1 Lineberger Comprehensive Cancer Center, University of North Carolina at Chapel Hill, Chapel Hill, North Carolina, United States of America; 2 Genetics Department, University of North Carolina at Chapel Hill, Chapel Hill, North Carolina, United States of America; 3 Department of Medicine, Division of Hematology and Oncology, University of North Carolina at Chapel Hill, Chapel Hill, North Carolina, United States of America; 4 Now at Baylor Sammons Cancer Center, Dallas, Texas, United States of America; 5 Department of Pharmacology and Cancer Biology, Duke University Medical Center, Durham, North Carolina, United States of America; 6 Now at Department of Cell Biology, Harvard Medical School, Boston, Massachusetts, United States of America; Duke University, United States of America

## Abstract

**Background:**

Hypoxia Inducible Factors (HIF1α and HIF2α) are commonly stabilized and play key roles related to cell growth and metabolic programming in clear cell renal cell carcinoma. The relationship of these factors to discretely alter cell metabolic activities has largely been described in cancer cells, or in hypoxic conditions, where other confounding factors undoubtedly compete. These transcription factors and their specific roles in promoting cancer metabolic phenotypes from the earliest stages are poorly understood in pre-malignant cells.

**Methods:**

We undertook an analysis of SV40-transformed primary kidney epithelial cells derived from newborn mice genetically engineered to express a stabilized HIF1α or HIF2α transgene. We examined the metabolic profile in relation to each gene.

**Results:**

Although the cells proliferated similarly, the metabolic profile of each genotype of cell was markedly different and correlated with altered gene expression of factors influencing components of metabolic signaling. HIF1α promoted high levels of glycolysis as well as increased oxidative phosphorylation in complete media, but oxidative phosphorylation was suppressed when supplied with single carbon source media. HIF2α, in contrast, supported oxidative phosphorylation in complete media or single glucose carbon source, but these cells were not responsive to glutamine nutrient sources. This finding correlates to HIF2α-specific induction of Glul, effectively reducing glutamine utilization by limiting the glutamate pool, and knockdown of Glul allows these cells to perform oxidative phosphorylation in glutamine media.

**Conclusion:**

HIF1α and HIF2α support highly divergent patterns of kidney epithelial cell metabolic phenotype. Expression of these factors ultimately alters the nutrient resource utilization and energy generation strategy in the setting of complete or limiting nutrients.

## Introduction

Clear cell renal cell carcinoma (ccRCC) is the most common subtype of renal cell carcinoma (RCC) making up over 70% of RCC cases. ccRCC is considered to arise from cells of the renal tubule epithelium, and the majority of ccRCC cases contain inactivation of the tumor suppressor gene, von Hippel-Lindau (*VHL*), either by mutation or deletion [1], [2]. VHL protein (pVHL) acts as a tumor suppressor through its role as the substrate recognition protein in the E3 ubiquitin ligase complex, VBC [3].

pVHL is involved in numerous processes [4]–[6], but one of the most widely characterized is the regulation of a family of transcription factors, hypoxia inducible factors (HIFs), in an oxygen dependent manner [7]. Under normal oxygen conditions, prolyl hydroxylases (PHD1, 2, 3) add a hydroxyl group to proline residues on HIF1α (alpha) and HIF2α. These hydroxylated residues are recognized by the VBC complex, and both HIF factors are polyubiquitylated and rapidly degraded by the proteasome [8]. When pVHL is inactivated by mutation or deletion, HIFα subunits escape VBC mediated regulation and are stably present in the cytoplasm regardless of the oxygen state of the cell. They heterodimerize with the cognate partner, aryl hydrocarbon receptor nuclear transporter (ARNT), also known as HIFβ (beta). HIF α/β heterodimers translocate to the nucleus where they regulate transcription of target genes. Importantly, HIFα isoforms, HIF1α and HIF2α, can be distinctly expressed in both normal and cancer cells where they may exert specific effects on cell phenotype and metabolism that are not well understood. These effects are particularly uncertain in the case of HIF2α.

Hypoxia inducible factors HIF1 and HIF2 (referring to each α/β heterodimeric complex) are predicted to play roles in differentiation [9], [10], vasculogenesis [10], [11], cell growth [9], [12], [13], and metabolic function [14]. Aberrant expression or dysregulation of these factors can initiate transcriptional activation programs to ultimately create a more favorable environment for tumorigenesis. Efforts to clarify transcriptional targets have shown differential roles for HIF1 and HIF2 in gene target regulation [14], [15]. HIF1 is generally described as regulating genes important for metabolic, and in particular glycolytic, function [16]; while HIF2 has more often been observed to regulate cell growth and angiogenic functions, with few roles being attributed to cellular metabolism. However, the majority of studies designed to tease apart differential activities of HIF factors have revealed a high degree of tissue dependence on functional outputs [17].

Differential expression of HIF1 and HIF2 in ccRCC has been observed, with human tumors either expressing HIF1 and HIF2 or only HIF2, although the functional effect of the differing expression profiles on metabolic regulation are not fully understood [12]. Recent evidence suggests that the HIF1α locus is selectively lost in ccRCC tumors during progression to higher stages [18]. Carcinoma is increasingly being characterized as a metabolic disease based on the cancer cells’ dependence on various necessary nutrients from healthy surrounding cells [19], [20], or the dysregulation of metabolic machinery to ensure continued growth [21]. In particular, ccRCC tumors display unique metabolic features that define the cancer in genomic investigations [22], [23].

Little is known about the individual contributions of HIF1 and HIF2 in metabolic processes. Recently, HIF1, more than HIF2, in RCC cells has been found to promote a glutamine-dependent reductive carboxylation metabolic phenotype [24]. We sought to identify the metabolic effects of stable expression of HIF1 or HIF2 in renal-derived cells to reveal the differences and similarities in contributions to metabolic programming and nutrient utilization by these transcription factors.

## Results and Discussion

### Neonatal Epithelial Kidney Culture System Provides an In vitro Model to Study Genetically Engineered Expression of HIF1 and HIF2

**Figure 1:**
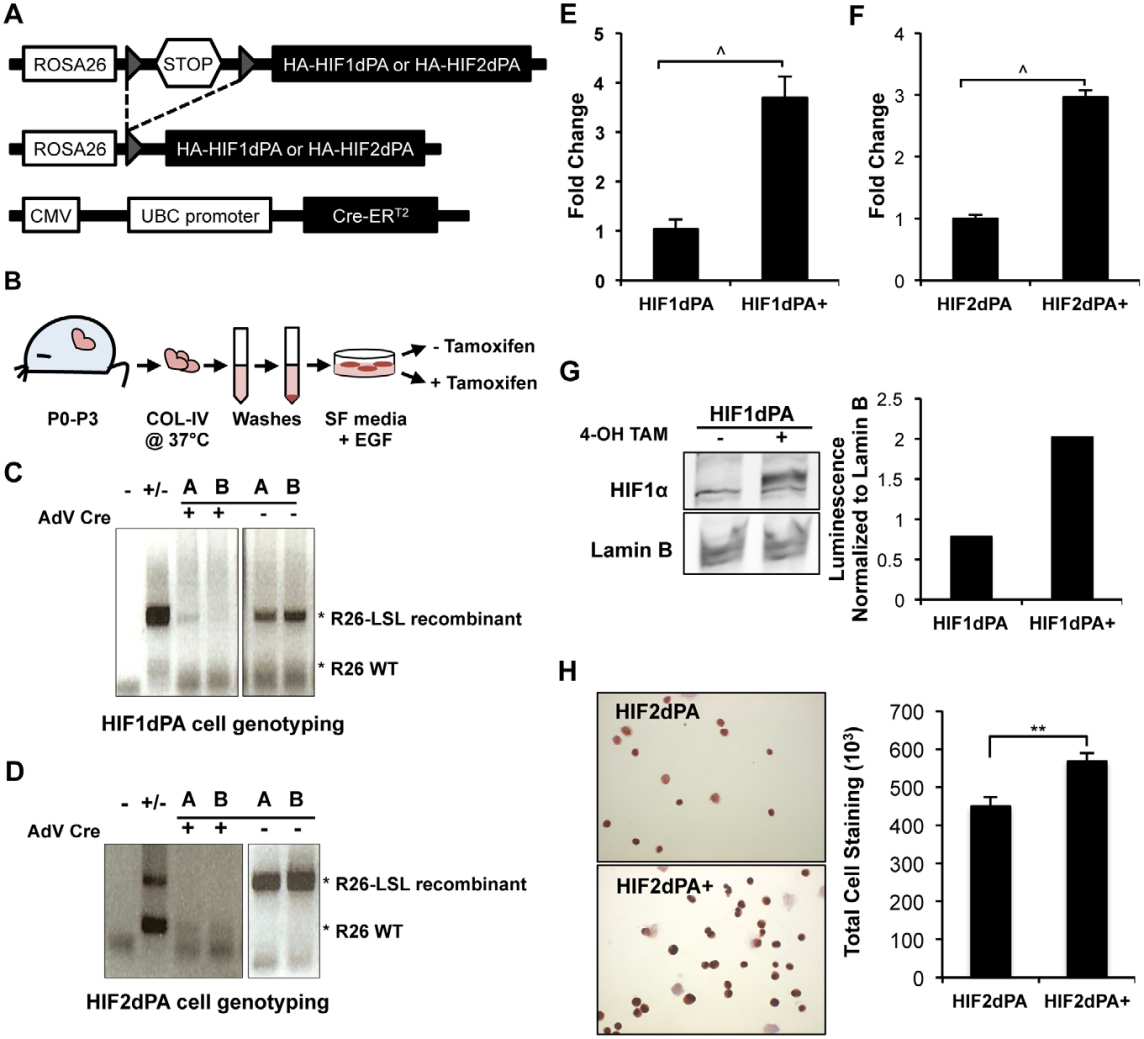
Conditional stable expression of Hypoxia Inducible Factor in a murine derived primary cell culture system. (**A**) The previously described transgenic mouse model [25] contains a HA-tagged human HIF1 or HIF2 insertion with double proline to alanine (dPA) substitutions in the endogenous Rosa 26 locus with inducible loxP-stop-loxP sites. These mice were crossed with a mouse containing a tamoxifen-inducible cre estrogen receptor ligand binding domain under control of a human ubiquitin C promoter [29]. Upon recombination, stable expression of HIF1dPA and HIF2dPA is induced. Recombination is denoted as “dPA+” throughout the paper. (**B**) Primary neonatal epithelial kidney cells were derived using both kidneys from a mouse 0–3 days post birth. The tissue was dissociated in collagenase IV and cultured in serum free (SF) media supplemented with EGF. NEK cells were recombined using 4-hydroxy tamoxifen (4-OHT). (**C**) Recombination and loss of the Rosa26-Lox-Stop-Lox alleles was confirmed in two newly isolated and recombined NEK HIF1dPA cells and (**D**) HIF2dPA cells by PCR primers specific to the R26LSL allele followed by gel electrophoresis. (**E**) qRT-PCR for the human *HIF1α* transcript was significantly increased in HIF1dPA+ cells. (**F**) Similarly, gene expression levels for the human *HIF2α* transcript was increased in HIF2dPA+ cells. (**G**) Immunoblot of nuclear extracts showed an increase in HIF1α expression following 4-OHT treatment and quantification of immunoblot for HIF1 protein expression displayed increased expression following recombination. Expression was normalized to an internal Lamin B control. (**H**) Immunocytochemistry for HIF2 protein expression in HIF2dPA+ cells compared to unrecombined HIF2dPA cells was used to quantify levels of staining across several areas. Corrected Total Cell Staining indicated significantly higher HIF2 protein levels in HIF2dPA+ cells. Bars indicate average with the SEM. **p≤0.01, ∧p≤0.001.

To focus on the differences in cellular function based on expression of HIFs independent of pVHL regulation, we employed a previously described transgenic mouse model of constitutive HIF dysregulation [25]. The conditional mouse model system contains a hemagglutinin (HA) tagged HIF1α subunit with a double proline to alanine substitution (dPA) at amino acid residues 402 and 564, here referred to as HIF1dPA; a separate mouse contains the HA-tagged HIF2α subunit with residues 405 and 531 containing proline to alanine substitutions, here referred to as HIF2dPA. Site-specific mutagenesis at these proline residues prevents hydroxylation by PHDs, thereby preventing polyubiquitination by the VBC complex and proteosomal degradation. Expression of these alleles is under control of the constitutively active Rosa26 promoter. Following recombination of loxP-stop-loxP (LSL) sites, HIFdPAs are stably expressed (Figure 1A).

Primary neonatal epithelial kidney (NEK) cell cultures [26], [27] (Figure 1B) were isolated using neonatal (0–3 days post birth) murine kidneys. Nephrogenesis, the development and growth of kidneys, continues up to postnatal day 3 and prior to this point the kidneys have not fully developed or terminally differentiated [28]. There remains an immature cell population that provides a platform for the exclusion of fibroblast cells and the promotion of epithelial cell growth by culturing the cells in serum-free media supplemented with epidermal growth factor (EGF).

Recombination of the R26-LSL alleles to activate expression of the HIFdPA was done by using the knock-in, 4-hydroxy tamoxifen (4-OHT) inducible ubiquitin C (UBC) Cre recombinase estrogen receptor 2 (Cre-ER^T2^) [29], allowing for complete recombination in all cell types (Figures 1C and 1D). Increased gene expression for the knock-in human *HIF1α* in HIF1dPA+ cells and *HIF2α* in HIF2dPA+ cells were confirmed by quantitative real time PCR (qRT-PCR) (Figures 1E and 1F). Confirmation of stable protein expression of HIF1α is demonstrated by immunoblot in HIF1dPA+ nuclear extracts (Figure 1G), and HIF2α in HIF2dPA+ cells by immunocytochemistry of cytospin preparations following recombination (Figure 1H). While these cells retain endogenous levels of HIF1α and HIF2α, they are normally expressed at low levels. Our data show through several independent techniques that this approach provides a basis for examining the individual effects of stably expressed HIF1α or HIF2α, in the form of a stable primary cell line derived from the murine kidney.

### Stable HIF Expressing Cells Differentially Activate Metabolic Target Genes

**Figure 2:**
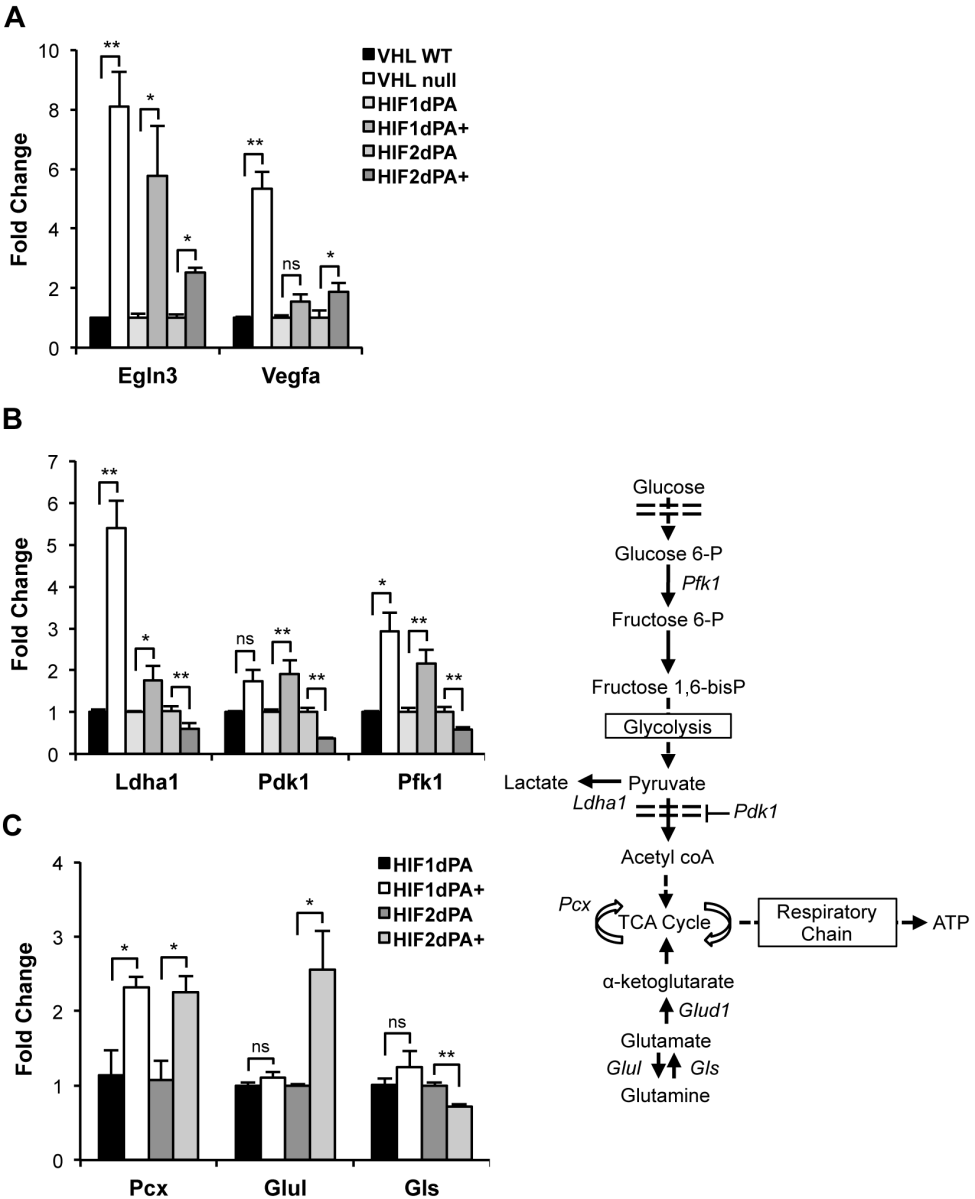
HIF1dPA and HIF2dPA are functional transcription factors. Gene expression of known HIF transcriptional targets was assessed by quantitative real time PCR. Fold change was calculated compared to the paired unrecombined cell line. (**A**) Gene expression of known joint targets *Egln3* and *Vegfa*. *Vhl* WT murine ES cells (endogenous HIFs lowly expressed) and *Vhl* null murine ES cells (endogenous HIFs highly expressed) show the increased expression of these targets when both HIF1 and HIF2 are expressed. Significant increase of *Egln3* expression in HIF1dPA+ and HIF2dPA+ cells, slight increase of *Vegfa* mRNA expression by HIF1dPA+, but significant increase of *Vegfa* by HIF2dPA+. (**B**) Expression of canonical HIF1 metabolic gene targets Lactate dehydrogenase (*Ldha1*), Pyruvate dehydrogenase kinase (*Pdk1*), and Phosphofructokinase (*Pfk1*). HIF1dPA+ cells have statistically significant increase in expression of *Ldha1*, *Pdk1* and *Pfk1*; HIF2dPA+ cells have decreased expression of the metabolic targets. (**C**) To assess the effect of HIF expression on metabolic gene expression, we analyzed gene expression by qRT-PCR of metabolic enzymes regulating entry into and progression of the TCA cycle; fold change of 4-OHT treated cells to paired unrecombined NEK cell line is shown. Both HIF1dPA+ and HIF2dPA+ cells had increased levels of Pyruvate carboxylase (*Pcx*) transcripts. HIF2dPA+ expressing cells showed increased levels of Glutamine synthetase (*Glul*) and decreased Glutaminase (*Gls*). Bars indicate average with the SEM. *p≤0.05, **p≤0.01, (ns) not significant.

HIF1 and HIF2 are known to regulate several common transcriptional targets, but independently are also capable of transcriptionally regulating specific target genes [14]. To assess the transcriptional function of the cell lines, qRT-PCR was performed for canonical HIF targets, egl nine homolog 3, (*Egln3*) [30] and vascular endothelial growth factor (*Vegfa*) [31]. Murine embryonic stem (ES) cells expressing a construct with WT *Vhl* [32] that exhibit maximal HIF regulation and *Vhl* null ES cells, where both HIFs are endogenously stabilized, were employed as controls. As expected, ES *Vhl* null cells had significantly elevated mRNA levels over *Vhl* WT cells for both HIF targets. A significant elevation in transcript levels of *Egln3* by both HIF1dPA+ and HIF2dPA+ cells was also observed. HIF1dPA+ cells only showed a slight increase in *Vegfa* mRNA levels, but a significant increase was observed in HIF2dPA+ cells (Figure 2A), consistent with previous reports suggesting that *Vegfa* responds preferentially to HIF2 in mouse models [33].

HIF1 has been regarded as a metabolic regulator by its known transcriptional regulation of various metabolic targets including the glucose transporter (*Glut1*); phosphofructokinase (*Pfk1*); lactate dehydrogenase (*Ldha1*); and pyruvate dehydrogenase kinase (*Pdk1*), which inhibits pyruvate dehydrogenase and the conversion of pyruvate into acetyl CoA, thereby blocking carbon entry into the tricarboxylic acid (TCA) cycle from glycolysis. *Vhl* null ES cells display a significant increase over ES *Vhl* WT cells in *Ldha1* and *Pfk1* mRNA levels by qRT-PCR. HIF1dPA+ cells also showed significant increases in *Ldha1*, *Pfk1*, and *Pdk1* mRNA levels over the unrecombined partner cell line, HIF1dPA. HIF2dPA+ cells did not show similar increases, and in fact showed a modest reduction in transcript levels of the same targets compared to HIF2dPA control cells (Figure 2B). All results were confirmed in at least two independently derived NEK cell lines. This verified that in our cell system, HIF1 is capable of regulating expression of glycolytic enzymes at the transcript level.

To understand the transcriptional role HIF1dPA+ and HIF2dPA+ cells might play in other metabolic processes, we analyzed mRNA levels of several key enzymes regulating metabolic activity (Figure 2C). We compared pyruvate carboxylase (*Pcx*), which metabolizes pyruvate to oxaloacetate for anapleurotic support of the TCA cycle. Additionally, we analyzed enzymes that catabolize glutamate into glutamine (glutamine synthetase, *Glul*) and glutamine into glutamate (glutaminase, *Gls*) for entry into the TCA cycle. Both HIF1dPA+ and HIF2dPA+ cells had significantly higher mRNA levels of *Pcx*, supporting anaplerosis, a reaction to replenishing TCA cycle intermediates. HIF2dPA+ cells exhibited an increased expression of *Glul* mRNA expression, an enzyme driving the conversion of glutamate into glutamine, along with decreased levels of *Gls*, indicating that glutamine sources may be blocked from being effectively utilized in HIF2dPA+ cells.

Transcriptional regulation of these enzymes details a potential role for HIF isoform expression in exerting influence in several key metabolic transitions affecting nutrient utilization, TCA cycle enzymes, as well as glycolysis. Examination of the equivalent human metabolic enzyme gene sequences (Table 1) revealed at least one putative hypoxia response element (HRE) upstream of each transcriptional start site (TSS). Previous groups have shown that some HIF target genes are known to contain functional HRE binding sites several thousand base pairs upstream [34]. The putative HRE binding sites in these genes still require individual confirmation of direct binding to HIFs and functional validation as transcriptional elements.

**Table 1:** Putative Hypoxia Response Element binding sites of metabolic regulatory enzymes.

Gene	HRE sequence	Distance from TSS (bp)
Pyruvate carboxylase	**G**CGTG	>> −2769, −2089, −1954, −747
	**A**CGTG	> −7188, −5738
Glutamine synthetase	**G**CGTG	−3022, −2770, −2442, −1355
Glutaminase	**G**CGTG	−506

### Glycolytic Function Differs According to Stable HIF Expression

**Figure 3:**
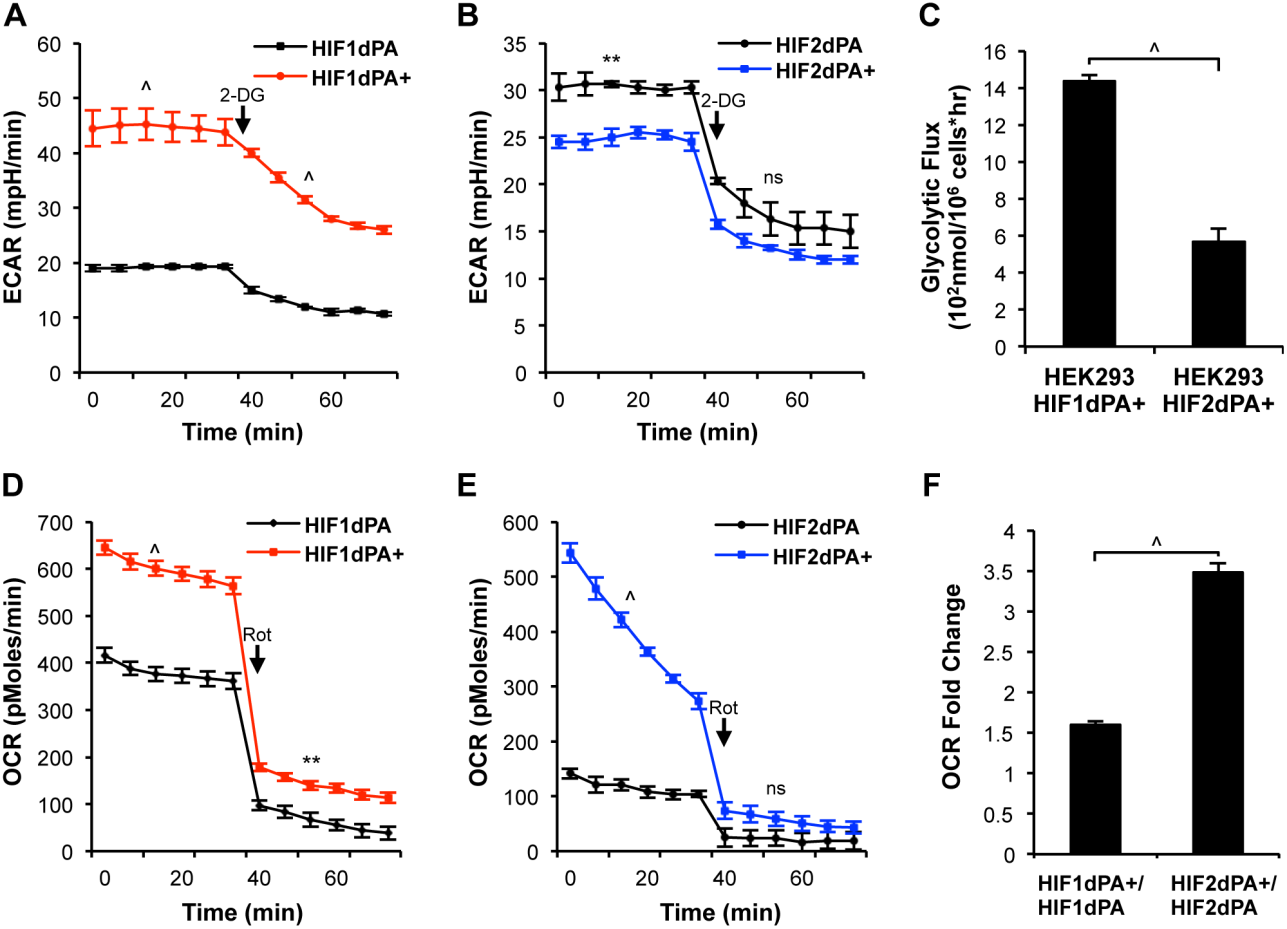
Metabolic function of differentially expressed HIF1 and HIF2. (**A**) Glycolytic function of NEK HIFdPA cells was quantified by measuring real time proton excretion, extracellular acidification rate (ECAR), using the Seahorse system. HIF1dPA+ cells incubated in complete media showed increased basal levels of glycolysis compared to HIF1dPA cells. Following treatment with a glycolytic inhibitor, 10 mM 2-deoxy-d-glucose (2-DG), levels are decreased, but a significant difference remains. (**B**) Basal and post treatment levels of glycolytic activity in HIF2dPA and HIF2dPA+ cells show no significant difference. (**C**) Glycolytic flux was measured in a separate cell line, human embryonic kidney cells (HEK 293) stably expressing either HIF1dPA or HIF2dPA. HEK HIF1dPA+ cells showed significantly increased levels of glycolytic flux. Results show the average of two independent runs. (**D**) Real time oxygen consumption rate (OCR) measurement in HIFdPA cells using the Seahorse system. HIF1dPA+ and (**E**) HIF2dPA+ cells have significantly higher levels of basal OCR over the paired unrecombined cell line. Following treatment with 750nM rotenone, an oxidative phosphorylation (OxPhos) inhibitor, OCR levels are greatly reduced. (**F**) Fold change in basal OCR levels show a significantly greater fold change in HIF2dPA+ over HIF2dPA, compared to the paired HIF1dPA+/HIF1dPA cells. Graphs indicate average with the SEM. **p≤0.01, ∧p≤0.001, (ns) not significant.

To explore the individual contribution of HIF1 and HIF2 to metabolic activity in kidney epithelia, we employed the Seahorse (XF) system to measure real-time extracellular metabolic flux and oxidative phosphorylation based on proton excretion and oxygen consumption, respectively [35]. HIF1 is well known to play a role in driving glucose uptake into cancer cell lines and enhancing glycolytic pathway activity [16], [36], but the effect of isolated HIF2 expression on glycolysis is not well known, or is predicted to be inconsequential. Primary NEK cells were cultured in complete media and examined for glycolytic activity via the extracellular acidification rate (ECAR) at basal levels and following the addition of a glycolytic inhibitor, 2-deoxy-d-glucose (2-DG). HIF1dPA+ cells displayed significantly increased basal levels of ECAR (red) compared to the control cell line, HIF1dPA (Figure 3A), as predicted. HIF2dPA+ cells (blue), in contrast, displayed a moderate reduction in basal levels of ECAR compared to unrecombined cells (Figure 3B), suggesting that HIF2 expression has a minor or potentially negative impact on production of lactic acid. Treatment with 2-DG, inhibited ECAR for both cell lines, confirming the measured acid production is derived primarily from glucose metabolism.

To verify that the difference in glucose utilization via glycolysis was not specific to one cell line or technique, we cultured an independent set of cell lines, human embryonic kidney (HEK 293) cells, induced for stable expression of either HIF1dPA+ or HIF2dPA+ with a radiolabeled glucose substrate to measure the amount of glucose metabolized and converted to radioactive water by the glycolytic enzyme enolase. HEK 293 HIF1dPA+ cells displayed significantly higher levels of glycolytic flux compared to HEK293 HIF2dPA+ cells (Figure 3C), confirming the preferential promotion of glycolytic function by HIF1 expressing cells.

### Both HIF Factors Contribute to Oxidative Phosphorylation in Complete Nutrient Environments

To assess whether HIF1 or HIF2 expression differentially influences oxygen consumption, as has been previously predicted [16], [37], [38], we measured oxygen consumption rate (OCR) by XF assay. Somewhat surprisingly, in complete media, both HIF1dPA+ (Figure 3D) and HIF2dPA+ cells (Figure 3E) demonstrated increased levels of basal oxidative phosphorylation (OxPhos) over the unrecombined paired cell line (black). Treatment with an OxPhos inhibitor, rotenone (Rot), which prevents complex 1 mitochondrial electron transfer thereby inhibiting the electron transport chain [39], resulted in reduced oxygen consumption, confirming the measured oxygen consumption is derived from oxidative phosphorylation activity. Thus, NEK cells stably expressing either HIF1 or HIF2 are capable of effecting increased mitochondrial oxidative phosphorylation activity. Previous observations of hypoxia-driven *Pdk1* expression had suggested that HIF1 transcriptional activation of *Pdk1* effectively blocked OxPhos [38]. Our results suggest that OxPhos can be induced by HIF1 expression, despite the increased levels of *Pdk1*. The fold increase in OxPhos in HIF1dPA+ cells compared to control, however, is less than half that of HIF2dPA+ cells, suggesting an effect of partial modulation or alternative pathways to TCA cycle activation (Figure 3F).

### Metabolic Pathway Preference Related to Carbohydrate Substrate Availability

**Figure 4:**
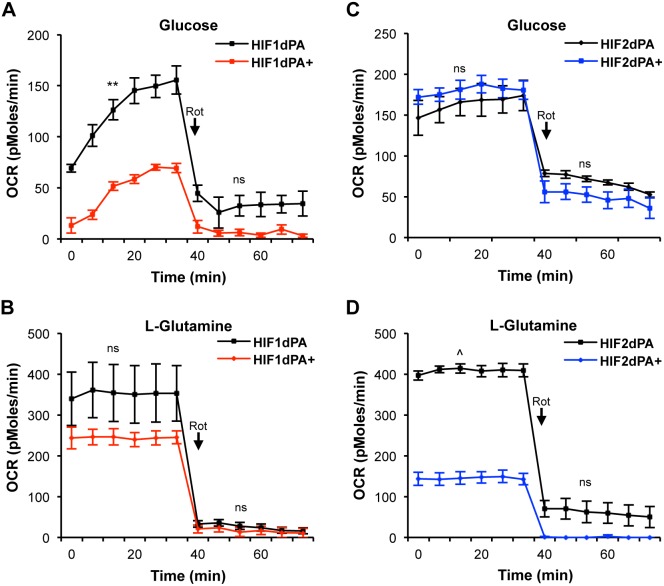
Differences in carbon source consumption and regulation of metabolic enzymes by differential HIF expression. Oxygen consumption rate (OCR) measurements before and after 750 nM Rotenone treatment of HIFdPA cells incubated in media supplemented with individual carbon sources. (**A**) HIF1dPA+ cells in 10 mM Glucose showed a significant decrease in OCR levels. (**B**) HIF1dPA+ cells displayed no difference in OCR with 2 mM L-glutamine. (**C**) HIF2dPA+ cells had no significant difference in OCR under glucose-supplemented conditions, (**D**) but significantly reduced OCR under L-glutamine-supplemented media. Lines indicate average with the SEM. **p≤0.01, ^∧^p≤0.001, (ns) not significant.

Cancer cells are capable of using various carbon sources to support cellular functions, with glucose and glutamine providing the majority of carbon nutrients [40]–[42]. To test nutrient contribution to energy production, we assayed the oxidative capabilities of NEK cells in the presence of media with only one major carbon source at a time. The cells were assayed in media with only glucose or L-glutamine as the main carbon source. HIF1dPA+ cells showed a significant decrease in oxygen consumption rate with glucose supplementation alone (Figure 4A), with relatively stable OxPhos when glutamine was the limiting carbon compared to control HIF1dPA cells (Figure 4B). This finding is consistent with prior studies suggesting a substantial shift toward aerobic glycolysis as the major metabolic feature of HIF1 expressing cells, and indicates that glycolysis may be the preferred method of energy production when nutrient carbons are limited, particularly to glucose. However, in conditions of unlimited nutrient resources, these cells are able to utilize a variety of metabolic processes, including OxPhos.

In contrast, HIF2dPA+ cells demonstrated slightly increased OCR levels over HIF2dPA control cells following the uptake of glucose alone (Figure 4C), but OCR levels were significantly suppressed in the presence of glutamine as a sole carbon source (Figure 4D). These results suggest that HIF2 expressing cells may utilize glucose as the preferred nutrient supporting mitochondrial OxPhos, but that glutamine as a sole carbon source is insufficient to support this process.

### Selective Carbon Metabolism is Influenced by HIF1-regulated Enzymes

**Figure 5:**
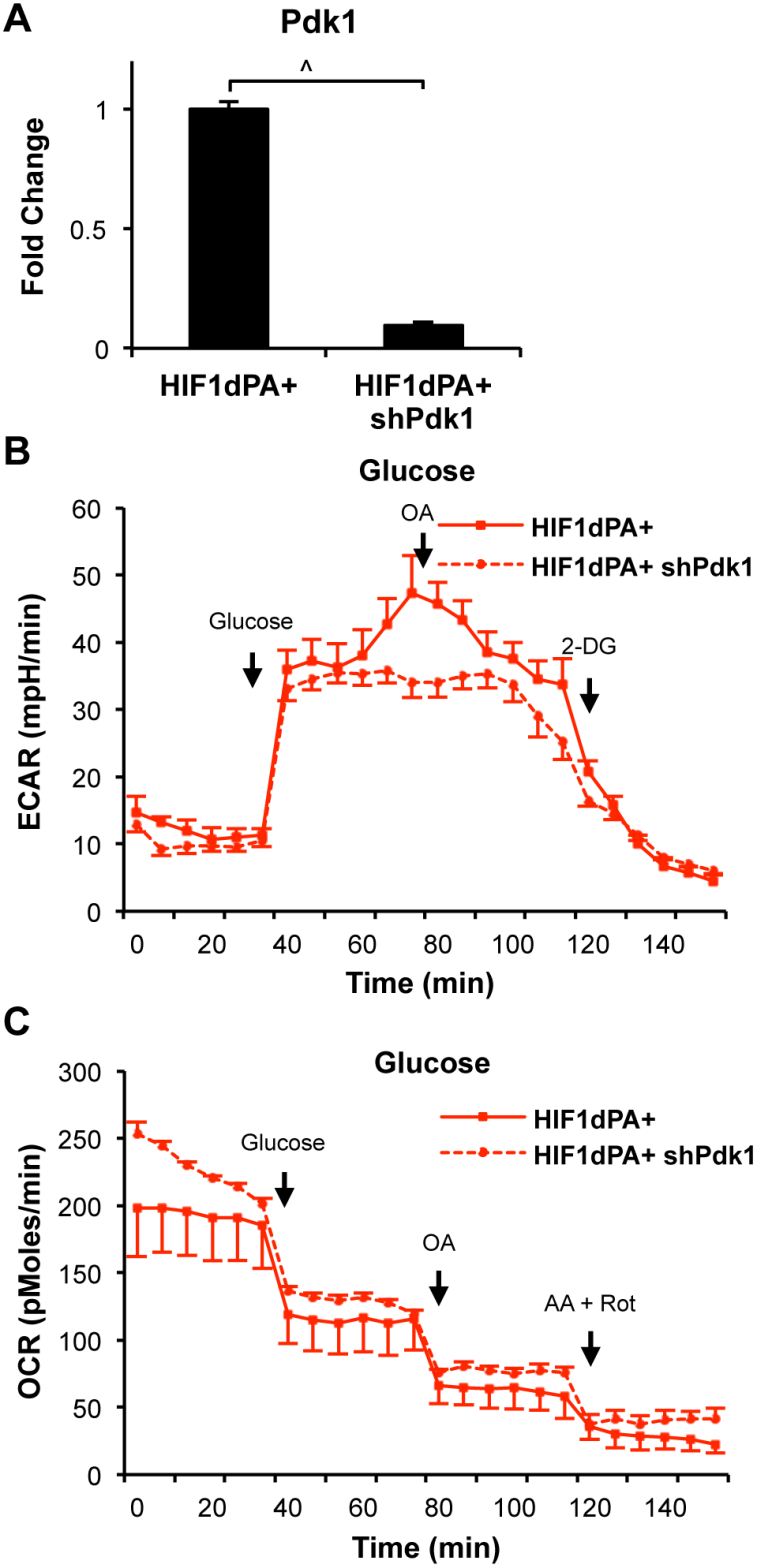
HIF1 regulation of glucose utilization dependent on Pdk1 expression. (**A**) qRT-PCR for the targeted transcript confirmed knockdown of *Pdk1* in HIF1dPA+ cells was ∼90% by shRNA. (**B**) HIF1dPA+ cells expressing shPdk1 showed decreased levels of ECAR following administration of 10 mM Glucose, even with the addition of 5 uM Oligomycin A. ECAR levels decreased upon 20 mM 2-DG treatment. (**C**) An increase in OCR levels in HIF1dPA+ shPdk1 cells was observed following Glucose treatment. OCR levels fell with the addition of 5 uM Oligomycin A and following 2 uM Antimycin A and 2 uM Rotenone co-treatment. Lines indicate average with the SEM. ∧p≤0.001.

The regulatory role of HIF1 on glycolysis is dependent on the initial uptake of glucose and increased expression of key enzymes of glycolysis. Secondarily, HIF1-dependent increases in *Pdk1* further promote lactic acid production by shuttling glycolytic substrates into lactate production. This has been suggested as a feature promoting survival in hypoxic settings [37]. The increased expression of *Pdk1*, primarily when induced in a hypoxic setting, has been shown to result in decreased oxygen consumption [38]. Because we had observed a HIF1-dependent increase in OxPhos in complete media, we wanted to assess how the levels of glycolytic acid production and oxygen consumption were influenced by *Pdk1* expression in this model system. We transfected HIF1dPA+ cells with a pool of short hairpin (sh) RNAs specific to *Pdk1* and confirmed knockdown efficiency at about 90% by qRT-PCR after 24 hours (Figure 5A). HIF1dPA+ shPdk1 cells were then assayed for ECAR and OCR levels following the addition of glucose. *Pdk1* knockdown cells showed a decreased media acidification response following glucose addition compared to HIF1dPA+ cells, which express the induced level of *Pdk1*. The difference is lost after 2-DG treatment confirming the effect is directly glucose dependent (Figure 5B). This result suggests that HIF1-induced increase in *Pdk1* contributes to the effective glycolytic production of lactic acid, likely via a component of diverting pyruvate away from the TCA cycle and promoting its conversion to lactic acid instead.

Additionally, we observed a modest, but measurable increase in oxygen consumption following glucose addition in *Pdk1*-deficient cells (Figure 5C). This finding suggests that the reduced levels of *Pdk1* would allow for more pyruvate to follow through to the TCA cycle and electron transport chain, resulting in a modest contribution to increased oxidative phosphorylation. Additionally, this finding and the reduced OxPhos observed in HIF1 expressing cells fed glucose alone, leaves open the possibility that alternative carbon sources may be contributing to the utilization of OxPhos by these cells.

### Glutamine Utilization by HIF2 is Dependent on Cellular Retention of Glutamate

**Figure 6:**
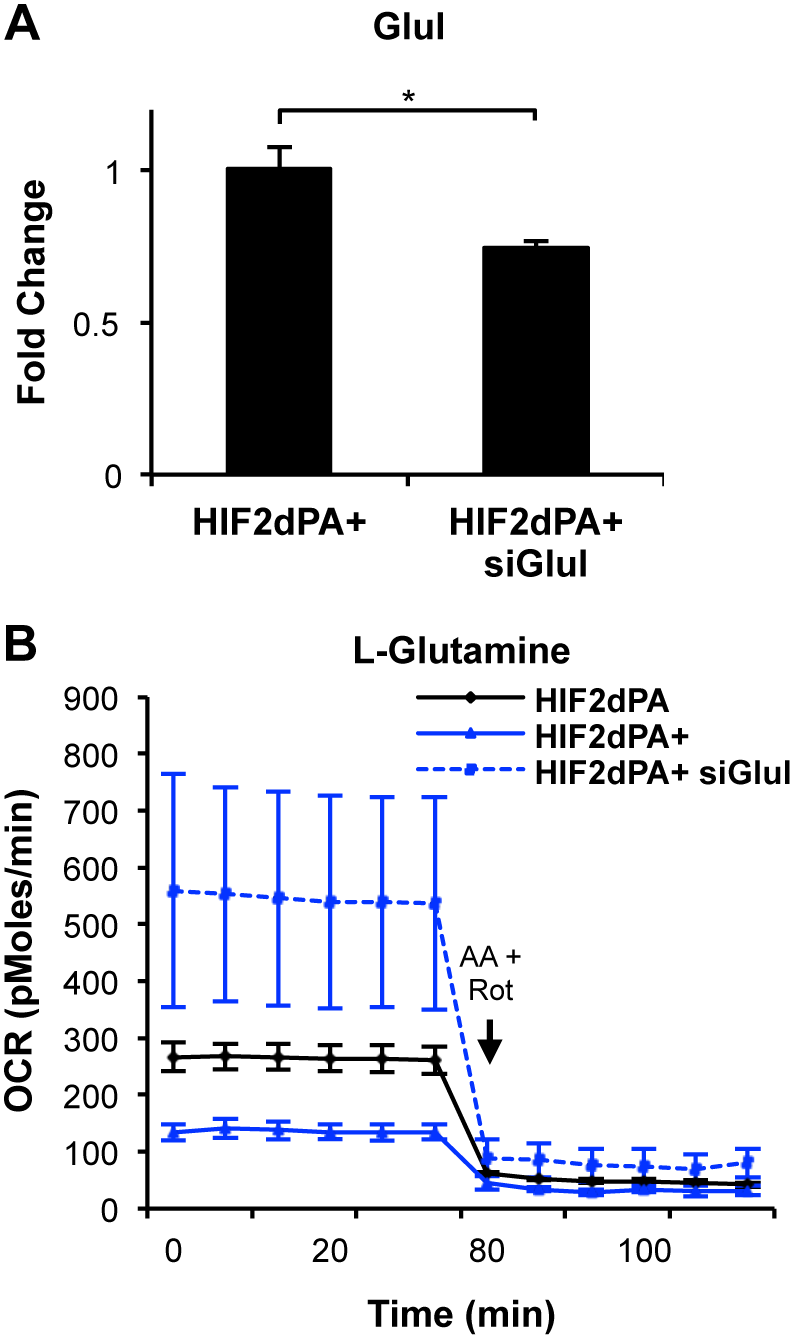
HIF2 glutamine utilization is deterred by Glul expression. (**A**) *Glul* knockdown by siRNA in HIF2dPA+ cells was ∼15% compared to untreated cells as confirmed by qRT-PCR. (**B**) Knockdown of *Glul* in HIF2dPA+ cells in 2 mM L-glutamine-supplemented media showed an increase in OCR over HIF2dPA+ cells. OCR levels decreased following 2 uM Antimycin A and 2 uM Rotenone co-treatment. Lines indicate average with the SEM. *p≤0.05.

The uptake of glutamine in cells can be regulated by several enzymes. As we previously described, HIF2dPA+ cells were less efficient at utilizing glutamine carbon source for OxPhos, and these cells displayed increased levels of *Glul* transcript, the enzyme required for glutamate to glutamine conversion. Increased expression of this enzyme would be predicted to reduce the available glutamate as an alternative substrate in the TCA cycle. We used a pool of four small interfering (si) RNAs to knockdown *Glul* in HIF2dPA+ cells. We confirmed knockdown (Figure 6A) by qRT-PCR compared to HIF2dPA+ cells. siGlul cells cultured in L-glutamine now demonstrated increased OxPhos levels (Figure 6B) compared to HIF2dPA+ cells, suggesting that this enzyme was creating a rate-limiting step in the utilization of glutamine for energy production. The OCR signal was quenched by inhibitors of electron transport chain complex I (Antimycin A and Rotenone), confirming that the signal reflected oxidative phosphorylation. Thus, while HIF2 cells are able to drive oxidative phosphorylation in the presence of complete media, and can maintain OxPhos energy production at wild type levels with glucose as a sole carbon source, their ability to metabolize glutamine is limited by the upregulation of *Glul*. Further, we observed a significant decrease in *Gls*, the enzyme that converts glutamine to glutamate, in the HIF2 expressing cells, which may further limit the utilization of glutamine as a resource to replenish TCA cycle substrate levels.

Taken together, we have observed that in renal primary epithelial cells, substrate availability for the TCA cycle is regulated by at least two nutrient pathway-specific points, pyruvate derived from glucose utilization and glycolysis, and glutamate derived from glutamine. HIF1 preferentially drives glucose metabolism, but can support the TCA cycle and oxidative phosphorylation when nutrient sources are unlimited, likely through both glutamine uptake and conversion to glutamate, as well as pyruvate conversion to acetyl coA. However, when nutrients are limited, glucose is preferentially utilized for glycolysis and the produced pyruvate is primarily shuttled into lactic acid production, as a result HIF1 is unable to support oxidative phosphorylation as nutrients become limiting. HIF2, in contrast, has minimal influences on glycolysis, but effectively supports oxygen consumption in nutrient rich environments, using primarily glucose, but not glutamine as a carbon source for substrate production. The limitation on glutamine utilization is at least partly driven by transcriptional upregulation of *Glul*, resulting in the shuttling of glutamate away from the TCA cycle. Knockdown of *Glul* releases this blockade, and permits highly effective oxidative phosphorylation using glutamine as a carbon source.

## Conclusions

Utilizing an endogenous HIF1 and HIF2 stable expression system in primary kidney cells, we were able to show that these related transcription factors have very different roles in the regulation of essential metabolic pathways. HIF1 has a greater role in regulating expression of key glycolytic enzymes and this effect is observed as an increase in glucose consumption and glycolytic acidification, but in nutrient rich conditions, HIF1 can support increased oxidative phosphorylation using a variety of TCA substrates. In contrast, HIF2 expression selectively increased oxygen consumption, and favored nutrient utilization of glucose. This preference appears to be due to a selective shuttling of TCA substrate glutamate back to glutamine, effectively limiting the ability of these cells to utilize glutamine as a carbon for energy production. The advantage of this blockade to cells is not clear. However, these findings suggest that HIF1 and HIF2 have preferential influences on metabolic activities, but that these energetic processes are dynamic and highly dependent on nutrient availability.

## Materials and Methods

### Ethics Statement

All animal work was done in accordance with the University of North Carolina at Chapel Hill Division of Laboratory Animal Medicine and was approved by the University of North Carolina at Chapel Hill Institutional Animal Care and Use Committee. (Protocol #12–195).

### Primary Cell Isolation and Culture

Transgenic mice containing human UBC-CreER^T2^ allele (Stock #008085) were purchased from JAX laboratories [9]. R26-LSL, HIFdPA mice were generously provided by Dr. William Y. Kim [25]. Animals were maintained on a standard chow. Adapted from previously published protocols [26], [27], bilateral kidneys were harvested from neonatal mice one to three days after birth. Both kidneys were rinsed in Dulbecco’s Phosphate Buffered Saline (D-PBS) twice. Tissues were crushed using plastic pestles and dissociated in 1 mg/mL Collagenase IV (Worthington) at 37°C for 40 minutes with intermediate vortexing. Cell/tissue suspension was washed in DMEM/F12 (Gibco) media twice to separate, by gravity, large remaining tissue pieces from cell suspension. The remaining cell suspension was washed in DMEM/F12 media and centrifuged at 500 rpm several times. Cells were cultured in NEK media at 37°C, 5%CO_2_. Cells were immortalized by treatment with 6 ug/mL polybrene in filtered conditioned media from ψ_2_SV40 cell culture, for 48 hours. The cells were subsequently cultured as an immortalized cell line. Cell cultures were recombined using NEK media containing 1×10^6^ units/mL Adenovirus Cre (University of Iowa, vector lab) for 48 hrs (for initial recombination) or 0.1 mM Z-4-hydroxytamoxifen (4-OHT) (Sigma) for 72 hours.

Cells were cultured in NEK media: DMEM/F12 base media, Insulin Transferrin Selenium (Gibco), Penicillin/Streptomycin (Gibco), 12 ng/mL human Epidermal Growth Factor (Sigma, Gemini Bioproducts).

### Cell Genotyping

Genomic DNA was extracted from cell suspension, by incubating a small cell pellet with 20 mg/mL Proteinase K solution at 55°C for 3 hours followed by 95°C for 15 mins. gDNA was then used to genotype each sample for the R26-LSL recombinant allele by polymerase chain reaction (PCR). PCR product was run on a 3% TAE agarose gel.

### Quantitative Real Time Polymerase Chain Reaction

cDNA was generated using SuperScript II Reverse Transcriptase (Invitrogen). qRT-PCR was performed using primer pairs designed using the online IDT Realtime PCR Scitools; primer sequences available upon request. The Maxima SYBR Green/Rox qPCR Master Mix (Thermo Scientific) and protocol were used for qRT-PCR. Samples were run on an Applied Biosystems Real-Time PCR Instrument. Sample cycle thresholds were normalized to Beta Actin. Fold change was calculated to the corresponding unrecombined cell line. Average fold change and standard error of the mean were graphed; Student’s two-tailed T-Test was performed to calculate significance at p≤0.05, p≤0.01, or p≤0.001.

### Quantitative Infrared Western Blot

Nuclear extractions of cells were conducted using the NE-PER Nuclear/Cytoplasmic Extraction Kit protocol (Pierce). ∼50 ug of protein from the nuclear extracts were run on a SDS-PAGE gel, transferred onto nitrocellulose membranes at 4°C, and washed in PBS-T. The membranes were blocked in 5% BSA in PBS, followed by incubation in 1∶1000 Rb anti-HIF-1α (Novus)/1∶500 Gt anti-Lamin B (Santa Cruz) solution in 5% BSA in PBS overnight at 4°C. The membranes were incubated in 1∶10,000 anti-Rb and anti-Gt secondary antibody at two different infrared channels in 5% BSA in PBS. The membranes were visualized on an Odyssey classic instrument; quantification of band intensity was done using Odyssey LI-COR software. Fold change of infrared fluorescence readings was done for HIF1 over nuclear Lamin B.

### Immunocytochemistry

200 uL of a 5×10^5^ cell suspension was used to cyto-spin cells onto a glass slide. The attached cells air-dried and were fixed in 3% Paraformaldehyde and permeabilized in a 0.25% Triton-X100 in PBS solution. Endogenous peroxidase activity was blocked by incubation in a 1% H_2_O_2_ solution. The samples were blocked in 5% BSA in PBS, then incubated in 1∶200 Rb anti-HIF-2α (Novus) primary antibody overnight in a humidified chamber. Secondary anti-rabbit antibody was added at 1∶10,000. Horseradish peroxidase (HRP) conjugation of the antigen in ABC-HRP solution (Vector kit) was followed by rapid incubation in DAB substrate solution (Vector Kit). Slides were counterstained with hematoxylin, dehydrated and coverslip mounted. Images were captured using an Olympus CX41 microscope with a mounted Infinity2 camera and Image Capture software. Stain intensity of individual cells was quantified using Image J software [43]. The average corrected total cell staining was graphed with standard error of the mean; Student’s two-tailed T-Test was performed to calculate significance at p≤0.01.

### Seahorse Metabolic Assay

Metabolic activity was measured using the Seahorse Biosciences XF24 Extracellular Flux Analyzer (Seahorse Biosciences) was used according to previously published methods [35]. 24-well Seahorse assay plate was coated with Cell-Tak (BD) to immobilize the cells. 4.5×10^4^ cells were plated in 24 well Seahorse assay plate (in replicates of 3–5), with triplicate blank wells to correct for the background. Cells were allowed to adhere at 37°C, 5% CO_2_. At the start of the experiment, the plated cells are washed in XF media with 10 mM Glucose added for complete nutrient media treatment. The sensor plate was hydrated overnight in XF calibrant media at 37°C and no-CO_2_, prior to calibration it was loaded with the indicated inhibitors or nutrients for a ten-fold dilution in the XF assay. Final concentrations used were 10 mM or 20 mM 2-deoxy-D-glucose (Sigma), 750 nM or 2 uM Rotenone (Ultra Scientific), 2 uM Antimycin A (Fisher Scientific), 10 mM Glucose (Fisher Scientific), 5 uM Oligomycin A (Fisher Scientific). Average fold change and standard error of the mean were graphed; Student’s two-tailed T-Test was performed to calculate significance at p≤0.05, p≤0.01, or p≤0.001.

XF assays with carbon-limited media were run using DME base media (D5030, Sigma, powder) dissolved in double processed water (W3500, Sigma) and brought to final concentration of 143 mM NaCl, Penicillin/Streptomycin (Gibco), with pH 7.1–7.3. For each limited carbon assay, the DME base media was brought to a final concentration of 10 mM Glucose (Fisher Scientific) or 2 mM L-glutamine (Gibco).

### Knockdown Assays

Cells were plate on XF cell culture plates, as described above, in NEK media without antibiotic (NEK-P/S) and allowed to adhere for three hours at 37°C, 5%CO_2_. Pooled shPdk1 lentiviruses (Sigma) were used and a shEmptyVector lentivirus was used as a negative control. shRNAs in NEK-P/S media were placed on the attached cell cultures treated with polybrene. Pooled siRNAs (siGlul, siNS, siGAPD) (Thermo Scientific) were reconstituted to 5 uM concentration in ddH2O. siRNAs were prepared for transfection using the DharmaFECT transfection reagent according to the manufacturer’s protocol (Thermo Scientific). Cells were incubated with siRNA for 24 hours at 37°C, 5% CO_2_. Seahorse metabolic assay on knockdown cells was performed as previously described 24 hours post-transfection. Separate cells were treated for RNA extractions at 24 hours post-transfection, to confirm knockdown efficiency by qRT-PCR.

### Glycolytic Flux

This assay was performed as previously published [44]. Glucose flux was determined in 2×10^6^ viable cells by washing in PBS followed by incubation in glucose-free Kreb’s buffer for 30 min prior to addition of 10 µCi of D-[5-^3^H](N)-glucose (PerkinElmer, Wellesley, MA) and non–radio-labeled glucose to bring total glucose concentration to 10 mM prior to culture for 1 hour. Reactions were stopped by addition of an equal volume of 0.2 N HCl. [^3^H]H_2_0 was separated from [^3^H]glucose by evaporated equilibrium in a sealed environment. Levels of [^3^H]H_2_0 produced were measured on a scintillation counter, and glycolytic flux was calculated as described [44]. Average fold change and standard error of the mean were graphed; Student’s two-tailed T-Test was performed to calculate significance at p≤0.05, p≤0.01, or p≤0.001.
